# The impact of icodextrin on the outcomes of incident peritoneal dialysis patients

**DOI:** 10.1371/journal.pone.0297688

**Published:** 2024-03-29

**Authors:** I-Kuan Wang, Chan Ip Chan, Alfred Hsing-Fen Lin, Tung-Min Yu, Tzung-Hai Yen, Ping-Chin Lai, Chi-Yuan Li, Fung-Chang Sung

**Affiliations:** 1 Divisions of Nephrology, China Medical University Hospital, Taichung, Taiwan; 2 Department of Medicine, College of Medicine, China Medical University, Taichung, Taiwan; 3 Baxter Healthcare Ltd., Taipei, Taiwan; 4 Graduate Institute of Management, National Taiwan University of Science and Technology, Taipei, Taiwan; 5 Raising Statistics Consultant Inc, Taipei, Taiwan; 6 Division of Nephrology, Taichung Veterans General Hospital, Taichung, Taiwan; 7 Division of Nephrology, Chang Gung Memorial Hospital, Taipei, Taiwan; 8 Chang Gung University, College of Medicine, Taoyuan, Taiwan; 9 Department of Anesthesiology, China Medical University Hospital, Taichung, Taiwan; 10 Management Office for Health Data, China Medical University Hospital, Taichung, Taiwan; 11 Department of Health Services Administration, China Medical University College of Public Health, Taichung, Taiwan; 12 Department of Food Nutrition and Health Biotechnology, Asia University, Taichung, Taiwan; Warren Alpert Medical School of Brown University: Brown University Warren Alpert Medical School, UNITED STATES

## Abstract

**Objective:**

The aim of the study is to investigate the effects of icodextrin on the risks of death, technique failure and the first episode of peritonitis in peritoneal dialysis (PD) patients.

**Methods:**

From medical records of a medical center in Taiwan, a total of 725 newly diagnosed end-stage kidney disease patients receiving PD for at least 90 days from January 1, 2007 to December 31, 2018 were identified. These patients were grouped as 190 icodextrin users and 535 non-users. Users were defined as utilization of icodextrin for ≥ 50% of their PD duration. The use of icodextrin was considered a time-varying exposure in the Cox proportional hazard model. The risks of death, technique failure and the first episode of peritonitis were compared between two cohorts by the end of 2018.

**Results:**

Compared to the non-users, the icodextrin users had significant lower risks of mortality (6.5 vs.7.2 per 100 person-years; adjusted HR = 0.62, 95% CI = 0.42–0.91) and technique failure (12.7 vs. 15.2 per 100 person-years; adjusted HR = 0.61, 95% CI = 0.47–0.81), and the first peritonitis episode (5.0 vs. 17.0 per 100 person-years; adjusted HR = 0.22, 95% CI = 0.14–0.35). The risk of peritonitis reduced further in icodextrin users with diabetes and with cardiovascular disease.

**Conclusion:**

Icodextrin was associated with lower risks of mortality, technique failure, and the first episode of peritonitis.

## Introduction

Peritoneal dialysis (PD) is a well-established home-based renal replacement therapy. Compared to in-center hemodialysis (HD), it has several advantages including better preservation of residual renal function, a more gradual and continuous solute and fluid removal, minimal cardiac stress, lower medical costs, treatment flexibility, better quality of life and similar survival [1–3]. In spite of these benefits, risks of technique failure and peritonitis are higher in patients undergoing PD than in those undergoing HD patients [4, 5]. Both are major challenges in caring for PD patients.

The conventional PD solution is glucose-based dialysate, which is cheap, safe, easily available, and an effective osmotic agent. However, the solution contains lactate, high concentrations of glucose, and glucose degradation products (GDPs) and has a low pH value and high osmolality [6]. These non-physiological components in this dialysate are not only harmful to the local peritoneal membrane but also have detrimental systemic effects on PD patients [6]. The higher glucose load in the glucose-based solution is associated with increased risks of technique failure and death [7, 8].

Icodextrin is an iso-osmolar mixture of corn starch-derived high molecular weight glucose polymers, containing lactate and no glucose, with a low pH value and a low concentration of GDPs. This solution is more slowly absorbed than glucose by the peritoneal cavity, mainly through lymphatics, maintaining the generated colloid osmotic pressure for a longer dwell (8–16 hours) [6]. Compared to glucose-based dialysates, icodextrin offers benefits, such as more biocompatibility, favorable metabolic effects and better fluid management [9–12].

However, the results of studies about the impact of icodextrin on outcomes of PD patients are inconsistent [13–16]. The aim of this study was to investigate the impact of icodextrin on the risks of mortality, technique failure, and peritonitis in a cohort of incident Asian PD patients. The risks between patients with and without icodextrin treatment identified from medical records of patients cared at China Medical University Hospital, a tertiary medical center in central Taiwan, were compared.

## Methods

From medical records retrieved from January 1, 2007 to December 31, 2018, incident end-stage kidney disease (ESKD) patients aged more than 18 years on PD for at least 90 days were identified. The data were assessed for research purposes from July 9, 2019 to July 8, 2020. The records of all eligible patients were retrospectively reviewed for information on demographic data, medical history, underlying comorbid conditions, laboratory data and treatment measures. Cardiovascular comorbidity was defined as a history of coronary artery disease, congestive heart failure, or stroke. Icodextrin users were defined as patients utilizing this dialysate for ≥ 50% of their PD duration [14]. According to the regulations of the Taiwan’s National Health Insurance, icodextrin (Extraneal; Baxter Healthcare Corporation) could be prescribed once daily in patients with an HbA1c > 7.0%, required 2.5% or 4.25% dextrose solution in more than half of the daily exchanges, or were in high or high-average peritoneal membrane transporter status. However, not all patients meeting these criteria had received icodextrin. The prescription of icodextrin was up to medical staff’s discretion for enhancing ultrafiltration and improving glycemic control. Medical records were reviewed until transfer from PD to HD, renal transplantation, transfer to another hospital, death, or December 31, 2018, whichever came first. During the review period, events of death, technique failure, and the first episode of peritonitis were identified. This study was performed in compliance with guidelines of the Declaration of Helsinki. This retrospective observational study was approved by the Research Ethics Committee of China Medical University Hospital [CMUH103-REC2-070 (CR5)]. All data were de-identified and analyzed anonymously. Authors had no access to information that could identify individual participants during or after data collection.

### Statistical analysis

The baseline characteristics between patients with and without icodextrin treatment were compared and tested by Chi-square test for categorical variables and Student’s t test for continuous variables. The multivariate time-dependent Cox proportional hazards model was used to estimate the adjusted hazard ratio (HR) and 95% confidence interval (CI). Covariates were sex, age, diabetes, hypertension, cardiovascular disease, modality [automated peritoneal dialysis (APD) vs. continuous ambulatory peritoneal dialysis], peritoneal equilibrium test (PET) (high average /high vs. low/low average), total Kt/V, normalized protein nitrogen appearance (nPNA), albumin, hemoglobin and the year of dialysis initiation. The usage of icodextrin was considered as a time-varying exposure in the Cox model. Noticeably, the use of icodextrin was defined as utilization of this solution for ≥ 50% of the patient’s PD duration [14]. If patients died within 90 days after switching to HD, the death was attributed to PD and counted as a death event. Otherwise, transfer to HD, renal transplantation, transfer to other hospital for care, and being alive at the end of the study period (December 31, 2018) were censored for patient survival analysis. Technique failure was defined as transfer to HD for at least 30 days or death on PD [17, 18]. Renal transplantation, transfer to another hospital for care, and being alive at the end of the study period were censored for technique survival analysis. Since the definition of icodextrin user as utilization of this solution for >50% of their PD duration was arbitrary, further data analysis by defining the icodextrin user as cut-offs of more than 25%, 33%, 67% and 75% of PD duration. In the analysis of the risk of the first episode of peritonitis, as there was a significant disparity in the proportion of renal transplantations between the two cohorts, the risk of the first peritonitis episode was re-evaluated after excluding patients who underwent renal transplantation. Subgroup analysis was performed and stratified by sex, age, diabetes, modality, PET and cardiovascular disease with adjustment for covariates. All tests were 2-tailed and p <0.05 was considered statistically significant. Data analyses were conducted using SPSS 26 (IBM SPSS Inc, Chicago, Illinois).

## Results

A total of 725 incident PD patients were identified from the medical records, consisting of 190 icodextrin users and 535 nonusers (Table 1). There were more men with lower mean age in the users than non-users without significant difference. Hypertension and diabetes were more prevalent in icodextrin users than non-users. The high or high average transporter status was also higher in users. In addition, icodextrin users had lower renal Kt/V, nPNA, and serum albumin, and higher peritoneal Kt/V and glycated hemoglobin (HbA1c), and were more likely to initiate PD in the year of 2007–2010, compared to the non-users. The mean follow-up period was longer in icodextrin users than in non-users (3.9 ± 2.8 vs. 3.2 ± 2.6 years; p = 0.002). During the follow-up period, 217 patients were transferred to HD: 61 (32.1%) of the icodextrin group, and 156 (29.2%) of the non-icodextrin group. 172 patients died during the follow-up period: 56 (28.5%) of the icodextrin group, and 116 (21.7%) of the non-icodextrin group. 12 of 56 (21.4%) patients in the icodextrin group and 21 of 116 (18.1%) in the non-icodextrin group died within 90 days after switching to HD. In addition, 51 patients received renal transplantation: 5 (2.6%) of the icodextrin group, and 46 (8.6%) of the non-icodextrin group. 40 patients were transferred to another center during the follow-up period: 10 (5.3%) of the icodextrin group, and 30 (5.6%) of the non-icodextrin group.

**Table 1:** The baseline characteristics of patients on peritoneal dialysis compared between cohorts of icodextrin users and non-users.

	Use of icodextrin during follow-up		* *
Variable	Yes (N = 190)	No (N = 535)	*P*
Male	110 (57.9)	267 (49.9)	0.058
Age, year	54.2 ± 13.5	56.3 ± 14.7	0.086
Comorbidity			
Diabetes	129 (67.9)	186 (34.8)	<0.001
Hypertension	156 (82.1)	402 (75.1)	0.05
Cardiovascular disease	55 (28.9)	123 (23.0)	0.101
Liver cirrhosis	4 (2.1)	19 (3.6)	0.329
Gout	10 (5.3)	35 (6.5)	0.53
Cancer	4 (2.1)	15 (2.8)	0.605
Hepatitis B virus infection	25 (13.2)	58 (10.8)	0.389
Hepatitis C virus infection	12 (6.3)	39 (7.3)	0.652
Dialysis modality			0.615
APD	71 (37.4)	211 (39.4)	
CAPD	119 (62.6)	324 (60.6)	
PET^a^			<0.001
L/LA	34 (18.0)	217 (40.8)	
HA/H	155 (82.0)	315 (59.2)	
Total Kt/V^b^	1.95 ± 0.40	1.96 ± 0.42	0.738
Renal Kt/V^b^	0.54 ± 0.37	0.65 ± 0.44	0.002
Peritoneal Kt/V^b^	1.41 ± 0.33	1.31 ± 0.37	0.001
nPNA^b^	0.99 ± 0.26	1.07 ± 0.26	<0.001
Albumin (g/dL)	3.5 ± 0.5	3.6 ± 0.5	0.002
Hemoglobin (g/dL)	9.8 ± 1.4	10.1 ± 3.9	0.252
HbA1c^c^ (N = 307)	7.6 ± 1.6	7.0 ± 1.3	<0.001
Cigarette smoking^d^			0.314
Never	150 (83.3)	459 (87.8)	
Former	10 (5.6)	20 (3.8)	
Current	20 (11.1)	44 (8.4)	
Alcohol drinking^d^			0.388
Never	167 (92.8)	499 (95.4)	
Former	9 (5.0)	16 (3.1)	
Current	4 (2.2)	8 (1.5)	
Etiology of ESKD			<0.001
Diabetes	123 (64.7)	165 (30.8)	
Chronic glomerulonephritis	35 (18.4)	227 (42.4)	
Chronic tubulointerstitial disease	6 (3.2)	27 (5.0)	
Hypertension	15 (7.9)	65 (12.1)	
Adult polycystic kidney disease	4 (2.1)	14 (2.6)	
Obstructive uropathy	2 (1.1)	6 (1.1)	
Others	5 (2.6)	31 (5.8)	
Year of dialysis initiation			0.003
2007–2010	76 (40.0)	156 (29.2)	
2011–2014	67 (35.3)	180 (33.6)	
2015–2018	47 (24.7)	199 (37.2)	
Follow-up year	3.9 ± 2.8	3.2 ± 2.6	0.002

APD, automated peritoneal dialysis; CAPD, continuous ambulatory peritoneal dialysis; L/LA: low/low average; HA/H: high average/high; nPNA: normalized protein nitrogen appearance; HbA1C, glycated hemoglobin; ESKD, end-stage kidney disease

^a^4 missing data

^b^8 missing data

^c^Restricted on diabetic patients

^d^22 missing data

Data were presented as mean ± standard deviation or frequency (percentage).

Table 2 shows that risks of mortality, technique failure and the first peritonitis episode were all significantly lower in icodextrin users than in non-users, with the corresponding adjusted HRs of 0.62 (95% CI = 0.42–0.91), 0.61 (95% CI = 0.47–0.81), and 0.22 (95% CI = 0.14–0.35), respectively, for the user cohort compared to the non-users. The results based on various cut-offs of PD duration for defining icodextrin users were in general consistent with that of the primary analysis with defining icodextrin users as utilization of this solution for >50% of PD duration. Moreover, the higher cut-off of PD duration for defining icodextrin users was associated with additional risk reduction in all three outcomes (S1 Table). After excluding 51 patients who underwent renal transplantation, the results also indicated a beneficial effect of icodextrin on the risk of the first episode of peritonitis (S2 Table).

**Table 2:** Mortality, technique failure and peritonitis compared between icodextrin users and non-users.

	Number of	Number of	Total	Incidence*	Unadjusted analysis		Adjusted^#^	
Outcome	Patients	events	PYs	(95% CI)	HR (95% CI)	*P* value	HR (95% CI)	*P* value
Death								
Control	535	116	1819.0	7.2 (6.0–8.4)	Reference		Reference	
Icodextrin	190	56	628.1	6.5 (4.5–8.5)	0.85 (0.60–1.22)	0.384	0.62 (0.42–0.91)	0.015
Technique failure								
Control	535	251	1817.2	15.2 (13.4–17.0)	Reference		Reference	
Icodextrin	190	105	628.1	12.7 (10.0–15.5)	0.81 (0.63–1.04)	0.099	0.61 (0.47–0.81)	<0.001
The first episode of peritonitis^†^								
Control	553^†^	178	1381.4	17.0 (14.8–19.2)	Reference		Reference	
Icodextrin	172^†^	63	476.8	5.0 (3.0–7.1)	0.33 (0.21–0.50)	<0.001	0.22 (0.14–0.35)	<0.001

PYs, person-years; CI, confidence interval; HR, hazard ratio.

^#^Adjusted for sex, age, diabetes, hypertension, cardiovascular disease, modality (APD vs. CAPD), PET (HA/H vs. L/LA), total Kt/V, nPNA, albumin, hemoglobin and year of dialysis initiation.

*Number of events per 100 person-years

^†^There were 18 patients suffered from peritonitis before the initiation of icodextrin.

The further subgroup analyses examined whether the 3 study outcomes associated with the efficacy of icodextrin therapy varied by selected covariates (Tables 3–5). The beneficial effects on patient and technique survival were greater for male and younger users, and patients on APD or with high average/high transporter status. The adjusted HRs of developing the first episode of peritonitis were extremely lower for patients with diabetes (0.07, 95% CI = 0.03–0.16) and cardiovascular (0.06, 95% CI = 0.02–0.22) (Table 5).

**Table 3:** Mortality in patients on PD compared between icodextrin users and non-users by subgroup analyses.

	Use of icodextrin						
	Users (N = 190)		Non-users (N = 535)				
Subgroup	Number	Incidence	Number	Incidence	cHR	aHR^#^	*P* for
	of events	(95% CI)*	of events	(95% CI)*	(95% CI)	(95% CI)	interaction^#^
Sex							0.558
Male	30	6.6 (3.9–9.2)	51	6.7 (5.0–8.4)	0.94 (0.58–1.53)	0.58 (0.33–0.04)	
Female	26	6.5 (3.5–9.5)	65	7.7 (5.9–9.4)	0.81 (0.48–1.36)	0.72 (0.41–1.25)	
Age							0.661
<65 years	38	5.1 (3.2–7.0)	60	5.1 (3.9–6.3)	0.96 (0.61–1.49)	0.48 (0.29–0.80)	
≥65 years	18	14.6 (6.9–22.2)	56	14.0 (10.5–17.5)	0.96 (0.54–1.73)	0.67 (0.35–1.29)	
Diabetes							0.067
No	13	2.5 (0.7–4.3)	62	5.2 (3.9–6.4)	0.45 (0.21–0.98)	0.31 (0.13–0.71)	
Yes	43	9.8 (6.5–13.1)	54	12.5 (9.4–15.6)	0.76 (0.50–1.15)	0.77 (0.48–1.23)	
Modality							0.193
CAPD	43	9.3 (6.1–12.5)	90	8.8 (7.1–10.5)	0.98 (0.66–1.46)	0.72 (0.47–1.11)	
APD	13	3.2 (1.1–5.2)	26	4.5 (2.9–6.1)	0.72 (0.34–1.53)	0.58 (0.24–1.40)	
PET							0.963
L/LA	9	5.2 (0.6–9.7)	36	5.7 (3.9–7.4)	0.89 (0.35–2.26)	0.79 (0.28–2.23)	
HA/H	46	6.6 (4.4–8.8)	80	8.2 (6.5–9.9)	0.75 (0.51–1.10)	0.57 (0.37–0.88)	
Cardiovascular disease							0.768
No	32	4.7 (2.7–6.6)	67	5.3 (4.1–6.5)	0.84 (0.52–1.35)	0.55 (0.33–0.93)	
Yes	24	12.0 (6.6–17.5)	49	15.0 (11.0–18.9)	0.80 (0.47–1.34)	0.81 (0.44–1.49)	

PD, peritoneal dialysis; cHR, crude hazard ratio; aHR, adjusted hazard ratio; CI, confidence interval; CAPD, continuous ambulatory peritoneal dialysis; APD, automated peritoneal dialysis; L/LA: low/low average transporter status; HA/H: high average/high transporter status.

*Number of events per 100 person-years.

^#^The analysis was adjusted for sex, age, diabetes, hypertension, cardiovascular disease, modality (APD vs. CAPD), PET (HA/H vs. L/LA), total Kt/V, nPNA, albumin, hemoglobin and year of dialysis initiation.

**Table 4:** Technique failure in patients on PD compared between icodextrin users and non-users by subgroup analyses.

	Use of icodextrin						
	Users (N = 190)		Non-users (N = 535)				
Subgroup	Number	Incidence	Number	Incidence	cHR	aHR^#^	*P* for
	of events	(95% CI)*	of events	(95% CI)*	(95% CI)	(95% CI)	interaction
Sex							0.792
Male	63	13.7 (9.8–17.6)	129	16.6 (13.9–19.3)	0.81 (0.58–1.12)	0.51 (0.33–0.78)	
Female	42	11.5 (7.5–15.5)	122	13.9 (11.5–16.3)	0.79 (0.54–1.17)	0.70 (0.48–1.003)	
Age							0.246
<65 years	74	10.5 (7.8–13.3)	153	12.3 (10.5–14.2)	0.84 (0.62–1.13)	0.47 (0.33–0.65)	
≥65 years	31	24.9 (15.0–34.9)	98	24.5 (19.8–29.2)	0.97 (0.62–1.52)	0.87 (0.52–1.45)	
Diabetes							0.06
No	23	5.4 (2.6–8.1)	141	11.3 (9.5–13.2)	0.45 (0.26–0.76)	0.40 (0.23–0.69)	
Yes	82	18.7 (14.2–23.3)	110	25.2 (20.8–29.6)	0.71 (0.53–0.96)	0.70 (0.50–0.98)	
Modality							0.082
CAPD	77	17.1 (12.8–21.5)	179	17.1 (14.7–19.5)	0.93 (0.69–1.24)	0.73 (0.53–1.01)	
APD	28	7.4 (4.2–10.6)	72	11.8 (9.2–14.5)	0.65 (0.40–1.06)	0.42 (0.24–0.74)	
PET							0.946
L/LA	21	14.5 (6.9–22.1)	95	14.5 (11.7–17.3)	0.99 (0.57–1.74)	0.66 (0.35–1.23)	
HA/H	83	12.2 (9.3–15.2)	153	15.4 (13.1–17.7)	0.76 (0.57–1.01)	0.60 (0.44–0.82)	
Cardiovascular disease							0.685
No	68	10.9 (7.9–13.8)	177	13.3 (11.4–15.2)	0.79 (0.58–1.07)	0.58 (0.42–0.82)	
Yes	37	18.4 (11.7–25.1)	74	22.8 (17.8–27.7)	0.80 (0.52–1.22)	0.75 (0.46–1.23)	

PD, peritoneal dialysis; cHR, crude hazard ratio; aHR, adjusted hazard ratio; CI, confidence interval; CAPD, continuous ambulatory peritoneal dialysis; APD, automated peritoneal dialysis; L/LA: low/low average transporter status; HA/H: high average/high transporter status.

*Number of events per 100 person-years.

^#^The analysis was adjusted for sex, age, diabetes, hypertension, cardiovascular disease, modality (APD vs. CAPD), PET (HA/H vs. L/LA), total Kt/V, nPNA, albumin, hemoglobin and year of dialysis initiation.

**Table 5:** Peritonitis in patients on PD compared between icodextrin users and non-users by subgroup analyses.

	Use of icodextrin						
	Users (N = 172)		Non-users (N = 535)				
Subgroup	Number	Incidence	Number	Incidence	cHR	aHR^#^	*P* for
	of events	(95% CI)*	of events	(95% CI)*	(95% CI)	(95% CI)	interaction
Sex							0.863
Male	32	4.8 (2.2–7.4)	85	16.9 (13.8–20.0)	0.31 (0.17–0.55)	0.22 (0.11–0.42)	
Female	31	5.3 (2.2–8.5)	93	17.2 (14.1–20.2)	0.33 (0.18–0.61)	0.21 (0.11–0.39)	
Age							0.17
<65 years	49	5.4 (3.1–7.7)	127	15.8 (13.4–18.2)	0.37 (0.24–0.58)	0.26 (0.16–0.42)	
≥65 years	14	2.9 (-1.1–6.9)	51	20.8 (15.9–25.7)	0.13 (0.03–0.54)	0.10 (0.02–0.42)	
Diabetes							<0.001
No	20	8.4 (4.2–12.7)	121	13.2 (11.0–15.5)	0.64 (0.37–1.09)	0.58 (0.33–1.02)	
Yes	43	3.0 (1.0–5.0)	57	27.5 (22.1–32.8)	0.15 (0.07–0.29)	0.07 (0.03–0.16)	
Modality							0.197
CAPD	38	4.2 (1.7–6.7)	117	18.1 (15.3–20.9)	0.24 (0.13–0.44)	0.17 (0.09–0.33)	
APD	25	6.1 (2.8–9.4)	61	15.1 (11.7–18.6)	0.42 (0.23–0.76)	0.28 (0.14–0.54)	
PET							0.302
L/LA	10	7.5 (0.9–14.1)	68	14.4 (11.2–17.6)	0.51 (0.20–1.25)	0.27 (0.10–0.72)	
HA/H	52	4.6 (2.6–6.7)	107	18.3 (15.4–21.2)	0.29 (0.18–0.46)	0.18 (0.11–0.31)	
Cardiovascular disease							0.032
No	45	6.0 (3.4–8.6)	137	15.5 (13.2–17.8)	0.41 (0.26–0.64)	0.30 (0.18–0.48)	
Yes	18	2.4 (-0.3–5.0)	41	23.6 (17.7–29.5)	0.12 (0.04–0.38)	0.06 (0.02–0.22)	

PD, peritoneal dialysis; cHR, crude hazard ratio; aHR, adjusted hazard ratio; CI, confidence interval; CAPD, continuous ambulatory peritoneal dialysis; APD, automated peritoneal dialysis; PET, peritoneal equilibrium test; L/LA: low/low average transporter status; HA/H: high average/high transporter status.

* Number of events per 100 person-years.

^#^The analysis was adjusted for sex, age, diabetes, hypertension, cardiovascular disease, modality (APD vs. CAPD), PET (HA/H vs. L/LA), total Kt/V, nPNA, albumin, hemoglobin and year of dialysis initiation

The causes of death are shown in S3 Table. Cardiovascular disease and infection were the major causes of death. The causes of technique failure are shown in S4 Table. Death and peritonitis were the major causes of technique failure. There were no significant differences in the causes of death and technique failure between the two groups (P = 0.658 and 0.297, respectively.)

## Discussion

Our study demonstrated that PD patients using icodextrin were at lower risks of mortality, technique failure, and peritonitis. The subgroup analyses showed that icodextrin could confer additional advantages for lowering the risk of the first episode of peritonitis in those with diabetes and cardiovascular disease.

Icodextrin was introduced in the early 1990s as a glucose-sparing solution for long dwells [19]. Although icodextrin has several clinical benefits on PD care, studies on its impact on the technique and patient survival are limited. Several previous studies showed that icodextrin, as salvage therapy for fluid overload or ultrafiltration failure, could extend technique survival [20, 21]. A Korean retrospective observational study revealed that utilization of icodextrin was associated with lower risks of all-cause mortality (adjusted HR = 0.69, 95% CI = 0.53–0.90) and technique failure (adjusted HR = 0.60, 95% CI = 0.40–0.92) [14]. In this study, icodextrin users were defined as those who had utilized the solution for ≥ 50% of their PD duration [14]. However, information on important clinical data including membrane transport type, residual renal function, and adequacy data was unavailable. A randomized controlled trial among diabetic PD patients in Japan found that the technique survival rate was significantly better in icodextrin users than in non-users (71.4% versus 45.0%) [22]. However, this study was limited by a small sample size (41 patients). In a previous retrospective observational study, we reported that the icodextrin use could decrease technique failure and improve patient survival in incident PD patients, initiating dialysis from 2007 to 2011 [16]. An Australia and New Zealand study using multicenter registry data also found a lower risk of technique failure due to social reasons such as burnout in patients cared at centers with higher icodextrin usage [23]. A recent meta-analysis showed that icodextrin could increase ultrafiltration, reduce episodes of fluid overload, and probably minimize mortality risk. But it had no significant effects on technique failure, peritonitis, or residual renal function decline [13]. The study was limited by lack of long-term assessment, low mortality events (only 32 events) and variable trial qualities. These limitations factors may cause insufficient power to draw any reliable concluding remarks. Our study analyzed data from a large cohort of incident PD patients and could provide further evidence to support the benefits of icodextrin in both technique survival and patient survival.

Studies about the impact of icodextrin on the risk of peritonitis are limited. An Austria multicenter, longitudinal, prospective, observational study found no significant effects of icodextrin on peritonitis rates in incident and prevalent PD patients [15]. In the Peritoneal Dialysis Outcomes and Practice Patterns analysis, the likelihood of cure after a peritonitis episode increased with greater icodextrin use [24]. Our study demonstrated that icodextrin was associated with a reduced risk of the first episode of peritonitis in incident PD patients. The protective effect was more prominent for those with diabetes or cardiovascular disease.

The use of icodextrin has local peritoneal and systemic protective effects by minimizing glucose exposure and optimizing volume status. Icodextrin may preserve the peritoneal membrane function, have a positive influence on peritoneal host defense, and extend the PD duration because it contains low glucose, has a low GDP content and is iso-osmolar [6, 10, 25–27]. It can also enhance ultrafiltration in long dwells and mitigate fluid overload, especially in patients with high and high-average peritoneal transports [10, 11, 13]. The use of icodextrin therefore results in improved fluid balance and blood pressure control and reduction in left ventricle mass [28, 29]. The clearance of small solutes such as sodium and creatinine could be increased by icodextrin through enhanced convection [11]. In addition, icodextrin confers favorable metabolic control. The use of icodextrin could reduce weight gain and HbA1c level and improve dyslipidemia and insulin resistance [9, 12, 30–32]. Poor glycemic control is associated with subsequent risk of exit site, tunnel infection, technique failure, and mortality [33, 34].

The strength of this study is the use of a well-organized database of medical records collected in the recent decade with a sample size large enough to evaluate outcomes after a long follow-up period. There are limitations in this study. The number of exchanges that might influence the rate of peritonitis and medication use were not included in data analysis. Furthermore, this study was observational and retrospective in design. In addition, the prescription of icodextrin was up to physicians’ discretion, which might introduce selection and indication biases. However, multivariate analyses were performed to reduce these biases. This observational study contains valuable information that could be conveyed to the nephrology community to optimize the care of PD patients.

In conclusion, our study highlights the benefits of icodextrin over glucose-based dialysates. The use of icodextrin was associated with lower risks of mortality, technique failure, and the first episode of peritonitis. Further large-scale long-duration randomized controlled studies are necessary to validate our findings.

## Supporting information

S1 Table Risks of outcomes associated with icodextrin use by various cut-offs of PD duration for defining icodextrin users. (DOCX)

S2 Table Risk of the first episode of peritonitis compared between icodextrin users and non-users, by excluding 51 patients who underwent kidney transplantation. (DOCX)

S3 Table Death numbers and rates by causes compared between cohorts of icodextrin users and non-users. (DOCX)

S4 Table Technique failure numbers and rates by causes compared between cohorts of icodextrin users and non-users. (DOCX)
