# Supplementary material for: The impact of icodextrin on the outcomes of incident peritoneal dialysis patients
Source: PLoS One. 2024 Mar 29;19(3):e0297688. doi: 10.1371/journal.pone.0297688 (PMC10980222; doi:10.1371/journal.pone.0297688)
Supplement: S1 Table — (DOCX) [file pone.0297688.s001.docx]

Table S1. Risks of outcomes associated with icodextrin use by various cut-offs of PD duration for defining icodextrin users

|  |  | Unadjusted analysis | |  | Adjusted^#^ | |
| --- | --- | --- | --- | --- | --- | --- |
| Outcome / Definition | No. of icodextrin users | HR (95% CI) | *P* value |  | HR (95% CI) | *P* value |
| Death |  |  |  |  |  |  |
| >25% vs. Control | 224 | 1.03 (0.74–1.43) | 0.870 |  | 0.77 (0.53–1.12) | 0.168 |
| >33% vs. Control | 215 | 0.90 (0.64–1.27) | 0.558 |  | 0.68 (0.47–0.999) | 0.0497 |
| >50% vs. Control | 190 | 0.85 (0.60–1.22) | 0.384 |  | 0.62 (0.42–0.91) | 0.015 |
| >67% vs. Control | 157 | 0.64 (0.42–0.96) | 0.029 |  | 0.46 (0.30–0.71) | 0.001 |
| >75% vs. Control | 136 | 0.66 (0.44–1.01) | 0.054 |  | 0.47 (0.30–0.74) | 0.001 |
| Technique failure |  |  |  |  |  |  |
| >25% vs. Control | 224 | 0.96 (0.76–1.22) | 0.754 |  | 0.76 (0.59–0.99) | 0.041 |
| >33% vs. Control | 215 | 0.90 (0.70–1.14) | 0.373 |  | 0.71 (0.54–0.92) | 0.010 |
| >50% vs. Control | 190 | 0.81 (0.63–1.04) | 0.099 |  | 0.61 (0.47–0.81) | <0.001 |
| >67% vs. Control | 157 | 0.64 (0.48–0.85) | 0.002 |  | 0.49 (0.36–0.66) | <0.001 |
| >75% vs. Control | 136 | 0.62 (0.46–0.84) | 0.002 |  | 0.47 (0.34–0.65) | <0.001 |
| The first episode of  peritonitis^†^ |  |  |  |  |  |  |
| >25% vs. Control | 196 | 0.30 (0.20–0.46) | <0.001 |  | 0.20 (0.13–0.32) | <0.001 |
| >33% vs. Control | 189 | 0.30 (0.20–0.46) | <0.001 |  | 0.21 (0.13–0.33) | <0.001 |
| >50% vs. Control | 172 | 0.33 (0.21–0.50) | <0.001 |  | 0.22 (0.14–0.35) | <0.001 |
| >67% vs. Control | 148 | 0.35 (0.23–0.54) | <0.001 |  | 0.26 (0.16–0.40) | <0.001 |
| >75% vs. Control | 131 | 0.36 (0.23–0.56) | <0.001 |  | 0.27 (0.17–0.43) | <0.001 |

PD, peritoneal dialysis; CI, confidence interval; HR, hazard ratio.

^#^Adjusted for sex, age, diabetes, hypertension, cardiovascular disease, modality (APD vs. CAPD), PET (HA/H vs. L/LA), total Kt/V, nPNA, albumin, hemoglobin and year of dialysis initiation.

^†^Eighteen patients suffered from peritonitis before the initiation of icodextrin were excluded from the analysis.
