# Supplementary material for: The impact of icodextrin on the outcomes of incident peritoneal dialysis patients
Source: PLoS One. 2024 Mar 29;19(3):e0297688. doi: 10.1371/journal.pone.0297688 (PMC10980222; doi:10.1371/journal.pone.0297688)
Supplement: S2 Table — (DOCX) [file pone.0297688.s002.docx]

Table S2. Risk of the first episode of peritonitis compared between icodextrin users and non-users, by excluding 51 patients who underwent kidney transplantation

|  | Number of | Number of | Total | Incidence* | Unadjusted analysis | |  | Adjusted^#^ | |
| --- | --- | --- | --- | --- | --- | --- | --- | --- | --- |
|  | patients | events | PYs | (95 % CI) | HR (95% CI) | *P* value |  | HR (95% CI) | *P* value |
| Control | 489 | 165 | 1265.4 | 11.3 (9.5–13.2) | Reference |  |  | Reference |  |
| Icodextrin^†^ | 167 | 61 | 461.0 | 5.2 (3.1–7.3) | 0.33 (0.21–0.50) | <0.001 |  | 0.23 (0.15–0.36) | <0.001 |

PYs, person-years; CI, confidence interval; HR, hazard ratio.

^#^Adjusted for sex, age, diabetes, hypertension, cardiovascular disease, modality (APD vs. CAPD), PET (HA/H vs. L/LA), total Kt/V, nPNA, albumin, hemoglobin and year of dialysis initiation.

^*^Number of events per 100 person-years

^†^Eighteen patients suffered from peritonitis before the initiation of icodextrin were excluded from the analysis.
