# Supplementary material for: The impact of icodextrin on the outcomes of incident peritoneal dialysis patients
Source: PLoS One. 2024 Mar 29;19(3):e0297688. doi: 10.1371/journal.pone.0297688 (PMC10980222; doi:10.1371/journal.pone.0297688)
Supplement: S3 Table — (DOCX) [file pone.0297688.s003.docx]

Table S3. Death numbers and rates by causes compared between cohorts of icodextrin users and non-users

|  |  | Use of icodextrin | |  |
| --- | --- | --- | --- | --- |
| Causes | Total  (N = 172) | Yes  (N = 56) | No  (N = 116) | *P* |
| Cardiovascular | 93 (54.1) | 33 (58.9) | 60 (51.7) | 0.658 |
| Infection | 50 (29.1) | 15 (26.8) | 35 (30.2) |  |
| Others^#^ | 29 (16.9) | 8 (14.3) | 21 (18.1) |  |

^#^Others (gastrointestinal bleeding, cancer, liver failure, respiratory failure, accident, other causes);

Data were presented as frequency (percentage).
