# Supplementary material for: The impact of icodextrin on the outcomes of incident peritoneal dialysis patients
Source: PLoS One. 2024 Mar 29;19(3):e0297688. doi: 10.1371/journal.pone.0297688 (PMC10980222; doi:10.1371/journal.pone.0297688)
Supplement: S4 Table — (DOCX) [file pone.0297688.s004.docx]

Table S4. Technique failure numbers and rates by causes compared between cohorts of icodextrin users and non-users

|  |  | Use of icodextrin | |  |
| --- | --- | --- | --- | --- |
| Reasons | Total  (N = 356) | Yes  (N = 105) | No  (N = 251) | *P* |
| Death on PD | 139 (39.0) | 44 (41.9) | 95 (37.8) | 0.208 |
| Peritonitis | 89 (25.0) | 21 (20.0) | 68 (27.1) |  |
| Burnout | 64 (18.0) | 15 (14.3) | 49 (19.5) |  |
| Medical problems | 29 (8.1) | 12 (11.4) | 17 (6.8) |  |
| Other PD-related problems^#^ | 35 (9.8) | 13 (12.4) | 22 (8.8) |  |

^#^Other peritoneal dialysis related problems (Inadequate dialysis, mechanical problems, exit site or tunnel infection, and encapsulated peritoneal sclerosis);

Data were presented as frequency (percentage).
